# Copy number variation contributes to cryptic genetic variation in outbreak lineages of *Cryptococcus gattii* from the North American Pacific Northwest

**DOI:** 10.1186/s12864-016-3044-0

**Published:** 2016-09-02

**Authors:** Jacob L. Steenwyk, John S. Soghigian, John R. Perfect, John G. Gibbons

**Affiliations:** 1 Biology Department, Clark University, 950 Main Street, Worcester, MA USA; 2 Current address: Department of Biological Sciences, Vanderbilt University, Nashville, TN USA; 3 Current address: Department of Environmental Sciences, The Connecticut Agricultural Experiment Station, New Haven, CT USA; 4 Division of Infectious Diseases, Department of Medicine, Duke University Medical Center, Durham, NC USA

**Keywords:** Cryptococcus gattii, Copy number variation, Pathogen, Pathogenicity, Population genomics

## Abstract

**Background:**

Copy number variants (CNVs) are a class of structural variants (SVs) and are defined as fragments of DNA that are present at variable copy number in comparison with a reference genome. Recent advances in bioinformatics methodologies and sequencing technologies have enabled the high-resolution quantification of genome-wide CNVs. In pathogenic fungi SVs have been shown to alter gene expression, influence host specificity, and drive fungicide resistance, but little attention has focused specifically on CNVs. Using publicly available sequencing data, we identified 90 isolates across 212 *Cryptococcus gattii* genomes that belong to the VGII subgroups responsible for the recent deadly outbreaks in the North American Pacific Northwest. We generated CNV profiles for each sample to investigate the prevalence and function of CNV in *C. gattii*.

**Results:**

We identified eight genetic clusters among publicly available Illumina whole genome sequence data from 212 *C. gattii* isolates through population structure analysis. Three clusters represent the VGIIa, VGIIb, and VGIIc subgroups from the North American Pacific Northwest. CNV was bioinformatically predicted and affected ~300–400 Kilobases (Kb) of the *C. gattii* VGII subgroup genomes. Sixty-seven loci, encompassing 58 genes, showed highly divergent patterns of copy number variation between VGII subgroups. Analysis of PFam domains within divergent CN variable genes revealed enrichment of protein domains associated with transport, cell wall organization and external encapsulating structure.

**Conclusions:**

CNVs may contribute to pathological and phenotypic differences observed between the *C. gattii* VGIIa, VGIIb, and VGIIc subpopulations. Genes overlapping with population differentiated CNVs were enriched for several virulence related functional terms. These results uncover novel candidate genes to examine the genetic and functional underpinnings of *C. gattii* pathogenicity.

**Electronic supplementary material:**

The online version of this article (doi:10.1186/s12864-016-3044-0) contains supplementary material, which is available to authorized users.

## Background

Copy number variants (CNVs) are a class of structural variants (SVs) and are defined as fragments of DNA, typically larger than 1 Kilobase (Kb), that are present at variable copy number in comparison with a reference genome [1]. Mutation rates of CNVs are typically higher than those of single nucleotide polymorphisms (SNPs) [2, 3] and several mechanisms have been proposed to explain the formation of CNVs, including non-allelic homologous recombination, non-homologous end-joining, retrotransposition, and fork stalling and template switching [4–7]. Recent developments in sequencing technologies and bioinformatics tools have made it possible to more accurately identify and quantify CNVs, revealing their prevalence in the genome [8–12]. For instance, estimates suggest CNVs encompass up to 10 % of the human genome, account for more base pair differences between individuals than single nucleotide polymorphisms (SNPs), and overlap hundreds of genes [13].

Copy number variation is an important source of both genotypic and phenotypic variation, and commonly exerts its functional consequences through the modulation of gene expression [14]. Copy number variation can influence gene expression and gene function through a number of molecular mechanisms including gene dosage, regulatory element dosage, gene interruption, gene fusion, and position effect [15]. In humans, CNVs are associated with numerous genomic disorders including cancers, and autism-related disorders [16–19]. Conversely, CNVs can also lead to adaptive phenotypes such as the diet driven evolution of salivary amylase (*AMY1*) gene copy number (CN) in human populations, increased drug resistance of *Plasmodium falciparum* through elevated *gch1* CN, and nematode resistance in soybean through increased CN of the multi-gene *Rhg1* locus [20–22].

In recent years, a number of studies have utilized high-throughput sequencing technologies to characterize the prevalence and function of CNVs in vertebrate populations [11, 23–26]. However, few studies have examined population-level patterns of CNVs in fungi and oomycetes despite the observation that SVs can play a role in host specificity, fungicide resistance, and degree of pathogenicity [27–30]. For example, multiple copies and modulated expression of the effector gene *Avr1a* in the oomycete *Phytophthora sojae* increases virulence and enables evasion of soybean immunity [27]. CNVs have also been demonstrated as playing a role in *Cryptococcus neoformans* virulence*.* Chromosomal CN variation in *C. neoformans* can lead to reduced fungicide susceptibility by increasing copy number of *ERG11,* a target of fluconazole, and *ARF1,* an ABC transporter linked to azole susceptibility [29].

More than 1 million individuals die annually as a result of invasive mycoses and the vast majority of these life threatening infections occur in immuno-compromised individuals [31]. However, *Cryptococcus gattii* recently emerged as a primary human and animal pathogen, capable of infecting immuno-competent individuals [32]. A series of prominent and deadly *C. gattii* outbreaks occurred in Vancouver Island in 1999 and subsequently spread to mainland British Columbia, Oregon, and Washington [32, 33]. The epidemiology of these outbreaks was surprising considering *C. gattii* was believed to be endemic to subtropical and tropical regions [34]. The isolation of *C. gattii* from soil and trees revealed the global, but patchy, presence of *C. gattii* and the existence of four major phylogenetic lineages (denoted VGI, VGII, VGIII and VGIV) [33, 35, 36].

Within the Pacific Northwest (PNW), infections have been predominantly caused by isolates belonging to the VGIIa, VGIIb, and VGIIc subgroups [33]. Genomic and phylogenetic analyses suggest the PNW VGII subgroups likely originated from South America and Australia [33, 34, 37, 38]. The PNW VGII subgroups exhibit low levels of genetic variation indicative of a recent clonal expansion [30, 33, 37]. Despite the high degree of genetic similarity between PNW isolates, VGII subgroups display pathological diversity. For instance, VGIIa and VGIIc isolates exhibit heightened virulence and more commonly present as pulmonary infections, compared with VGIIb isolates which are generally less virulent and maintain the classical clinical pathology of neurological dominance [32, 33, 37]. Better understanding the genetic basis underlying VGII range expansion, niche adaptation, and pathological differences between subgroups could aid in the prevention, control, and treatment of future *C. gattii* outbreaks.

We hypothesize that CN variation could rapidly generate genotypic diversity in *C. gattii* subgroups and this variation might contribute to phenotypic and pathologic differences between VGII subgroups. To address this hypothesis, we identified 90 VGII isolates from more than 200 publically available *C. gattii* whole-genome Illumina sequencing datasets and generated high-resolution genome-wide CNV profiles for each isolate (Additional files 1 and 2) [30, 33, 35, 39]. Within predominantly PNW isolates (one isolate from Caribbean, and one isolate from Australia), we (i) broadly characterized genomic patterns of CNVs, (ii) identified divergent CNVs between the VGII subgroups, and (iii) examined the functional associations of genes overlapping with CNVs.

## Methods

### Data mining and sequence read processing

Whole-genome Illumina data for *C. gattii* isolates were retrieved from the NCBI Sequence Read Archive using the SRA Toolkit (Additional file 1) [30, 33, 35, 39]. For all samples, *trim_galore* (http://www.bioinformatics.babraham.ac.uk/projects/trim_galore/) was used to remove residual adapter sequences and trim reads at bases with quality scores below 30. Trimmed reads shorter than 100 nucleotides (nt) were discarded. Quality and adapter trimmed read sets were then mapped against the *C. gattii* R265 reference genome (https://www.broadinstitute.org/annotation/genome/cryptococcus_neoformans_b/MultiHome.html) using the ‘sensitive’ preset parameters in *Bowtie2* [40]. Mapping outputs were converted to sorted bam format using *samtools* ‘view’ and ‘sort’ parameters [41]. We first calculated average genome-wide coverage for each sample by dividing the sum of the per-base nucleotide depth values, using the *samtools* depth function, by the *C. gattii* R265 genome size [41]. Because differences in coverage between samples can bias CNV estimates, we used *seqtk* (https://github.com/lh3/seqtk) to randomly subsample reads such that each sample was normalized to 10× coverage. We chose a 10× coverage in order to retain the majority of publicly available data, and to ensure reliable CN estimation [42]. Samples with average pre-normalized coverage values less than 10× were removed from analysis, resulting in a total of 212 isolates. These 10× coverage subsampled datasets were mapped against the reference genome, and sorted bam files were generated as described above. The 10× coverage datasets were used for SNP calling and CNV estimation.

### Inferring population structure

We performed population structure analysis to identify isolates belonging to the VGIIa, VGIIb, and VGIIc, subgroups. SNPs were conservatively called for each sample using *VarScan.v2.3.9*, requiring a minimum coverage of 8X and a SNP frequency of 100 % [43]. SNP sites were removed from a consensus whole population matrix when >10 % of the population harbored an ambiguously called base, or when minor allele frequency was <10 %. We subsampled the resulting matrix to ensure SNP sites were separated by >10 Kb (average distance markers ~13 Kb). The final matrix used for evolutionary analysis consisted of 1223 SNPs.

We employed several methods to infer the population structure of the 212 isolates. First, we used the Bayesian model-based software *Structure 2.3.4*, using the “admixture” ancestry model and the “allele frequencies are correlated among populations” frequency model [44]. We ran a burn-in period of 100,000 and a Markov Chain Monte Carlo (MCMC) of 200,000 generations for *K* = 1–13, where *K* represents the number of genetic clusters or populations. The optimal number of clusters was predicted by calculating the average log probability (Ln*P*(D)) of each *K* value and by calculating the rate of change in the Ln*P*(D) between successive *Structure* runs with different *K* values (*ΔK*) [44, 45]. Both statistics were calculated using *Structure Harvester* [46]. Because *Structure* assumes Hardy-Weinberg equilibrium and linkage equilibrium and natural populations often violate these assumptions, we also employed *Discriminant Analysis of Principal Components* (*DAPC*) in the *adegenet v1.3-1* package. *DAPC* is a non-model-based multivariate approach, to infer population structure [47]. The number of distinct populations was predicted using the “find.clusters” *k*-means clustering algorithm and by calculating the Bayesian Information Criterion (BIC) value for each *K* = 1–16. Predicting the optimal population number is often unclear and complex in panmictic natural populations. An alternative approach is to define the number of populations that are useful in describing the genetic data [47]. Using this approach, BIC values smaller than 1000 represent useful summaries of population structure [47].

When assigning individuals to subpopulations, we assigned isolates to clusters when cluster assignment between *Structure* and *DAPC* were in agreement*.* Isolates with membership coefficients <90 % were removed*.* Clusters were assigned a VGIIa, VGIIb, or VGIIc classification according to predominant clustering of subgroups previously identified by other studies [30, 33, 35, 39]. This classification scheme resulted in 35 VGIIa, 22 VGIIb, and 33 VGIIc isolates.

### Genome-wide copy number quantification

Whole supercontig CN variation can be detected through differences in the relative number of mapped reads. To estimate supercontig CN, a normalized mapped read value (NMRV) for each sample was determined by calculating the number of reads mapped per supercontig using the *samtools* ‘idxstats’ function [41]. The NMRV was calculated using only ten largest supercontigs which have an average length of 1,181,220 base pairs (bp). To estimate supercontig CN, the number of reads that mapped to each supercontig was calculated using the *samtools* ‘idxstats’ function and then divided by NMRV. Thus, relative read mapping serves as a proxy for CN events of larger sizes.

We used the *Control-FREEC* software for high-resolution CNV quantification [48]. Using the read depth approach, *Control-FREEC* additionally implements a sliding window and Least Absolute Shrinkage and Selection Operator (LASSO) approach for CNV detection [48–50]. Integer CN was estimated for each 100 bp non-overlapping window of the genome using the following parameters: window = 100, telocentromeric = 0, minExpectedGC = 0.33, and maxExpectedGC = 0.63. CN of high V_ST_ genes was calculated as the average copy number value of 100 bp windows that overlap with the gene of interest.

### Identifying divergent CNVs between VGII subpopulations

The V_ST_ measurement, derived from F_ST_, is used to identify loci that differentiate by CN between populations [9]. V_ST_ and F_ST_ consider how genetic variation is partitioned at the individual, population, and global levels and range from 0 (no population differentiation) to 1 (complete population differentiation). V_ST_ has proven useful in identifying CNVs under positive selection in genetically distinct populations [8, 25, 51]. We calculated V_ST_ between VGIIa, VGIIb, and VGIIc groups for each non-overlapping 100 bp bin in the genome using the following formula:

$$ {V}_{ST}=\frac{V_{total}-\raisebox{1ex}{$\left({\mathrm{V}}_{\mathrm{V}\mathrm{GIIa}}\times {\mathrm{N}}_{\mathrm{V}\mathrm{GIIa}}+{\mathrm{V}}_{\mathrm{V}\mathrm{GIIb}}\times {\mathrm{N}}_{\mathrm{V}\mathrm{GIIb}}+{\mathrm{V}}_{\mathrm{V}\mathrm{GIIc}}\times {\mathrm{N}}_{\mathrm{V}\mathrm{GIIc}}\right)$}\!\left/ \!\raisebox{-1ex}{${\mathrm{N}}_{\mathrm{total}}$}\right.}{V_{total}} $$

V_total_ is total variance, V_VGIIx_ is the CN variance for each respective subpopulation, N_VGIIx_ is the sample size for each respective subpopulation, and N_total_ is the total sample size. We considered V_ST_ values in the upper 99th percentile, corresponding to values greater than 0.3397, as significant.

### Genomic statistics

To assess differences in gene gain or loss between subgroups, we used a non-parametric Fisher-Pitman permutation test implemented in the R package *coin*, using 1,000,000 permutations [52]. The R package *boot* was utilized for bootstrapping statistics to provide confidence intervals with 1,000,000 bootstrap replicates [53, 54]. If statistical significance was observed between subgroups, permutation-based pairwise *T-tests* (100,000 permutations) with Bonferroni-corrected *p-values*, as implemented in the R package *RVAideMemoire,* were used to assess statistical differences between subgroups [55].

### Enrichment analysis

We used Gene Ontology (GO) annotation from the *C. neoformans* H99 genome to assign functional classification to *C. gattii* orthologs [56]. Orthologs were predicted using the reciprocal best-BLAST hit approach with an *e*-value cut-off of 1e^−6^ [57, 58]. PFam domains in high V_ST_ genes were predicted using *interproscan-5.8-49.0* [59]. Enrichment analysis was conducted using default settings in *GOEAST* (GO terms) and *dcGO* (PFam domains) [60, 61]. Results from both web-based programs were visualized in *REVIGO* [62].

## Results

### Population structure of *C. gattii* isolates

**Fig. 1:**
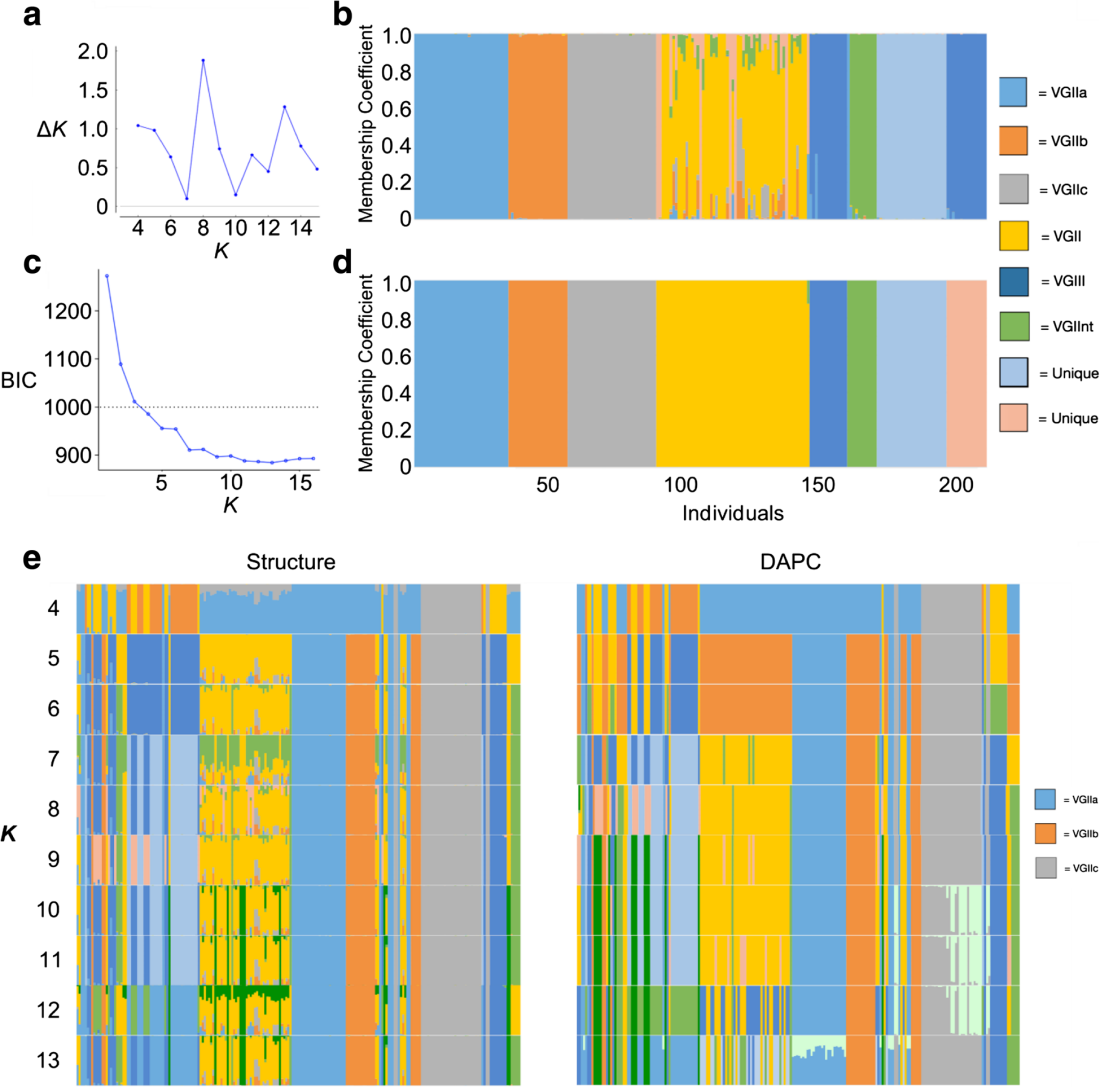
*Structure* (**a, b**) and *DAPC* (**c, d**) analyses of 212 *Cryptococcus gattii* isolates identify the presence of identical VGIIa, VGIIb, and VGIIc genetic clusters. **a** In the *Structure* analysis, the ∆*K* statistic predicts that the optimal number of populations (*K*) is eight. **b** *Structure*-based individual assignment of the 212 *C. gattii* isolates into eight populations. The Y-axis represents individual membership coefficient, while the X-axis corresponds to the 212 individuals. Thirty-five VGIIa, 22 VGIIb, and 33 VGIIc isolates were identified. **c** *DAPC* analysis indicates that values of *K* between 4 and 16 represent an informative number of populations. *K* values below the Bayesian information criterion (BIC) cutoff value (dotted line) represent useful summaries of population structure. **d** When *K* = 8 individual population assignment into the VGIIa, VGIIb, and VGIIc populations was identical compared with *Structure*. **e** Stability and agreement of individual population assignment into the VGIIa, VGIIb, and VGIIc subpopulations for *K* between 5 and 9 between *Structure* and *DAPC*. VGIIa, VGIIb, and VGIIc individuals are colored coded as *blue*, *orange*, and *grey*, respectively, through different values of *K*

We were primarily interested in identifying loci with distinct CN profiles between the VGII subpopulations. Thus, we first performed population structure analysis in order to identify VGIIa, VGIIb, and VGIIc isolates using *Structure* and *DAPC* [44, 47]. We generated a SNP matrix of 1223 informative loci for 212 individuals (see Methods), and determined that a *K* of eight was the optimal number of populations by the Δ*K* method (Fig. 1a) [45]. With the same SNP matrix, *DAPC* did not determine a single optimal *K* but rather an informative range of *K* from 4 to 16 (Fig. 1b) [47]. We chose a *K* value of 8 as it was the optimal value in *Structure* and fell within *DAPC’s* suggested range. Membership coefficients were independently calculated using *Structure* and *DAPC* (Fig. 1c and d, respectively) [44, 47]. When assessing membership coefficients across *K* 5–13, VGIIa, VGIIb and VGIIc individuals clustered together consistently (Fig. 1e). Using this approach, a total of 35, 22, and 33 VGIIa, VGIIb, and VGIIc isolates were identified, respectively (Fig. 1b, d, and e). Subsequent CN variation analysis was performed on this set of 90 VGII isolates.

### Copy Number Variant (CNV) analysis

We generated high-resolution CNV profiles for the 90 VGIIa, VGIIb, and VGIIc isolates to better understand the contribution of CNVs to genome variation. On average, CNV regions encompassed 1.63 % (284,860 bp) of the VGIIa genomes, 2.32 % (404,935 bp) of the VGIIb genomes, and 1.96 % (341,803 bp) of the VGIIc genomes, in comparison to the reference R265 genome. The average number of CNV regions per sample for VGIIa, VGIIb, and VGIIc was 156 ± 20.97, 171 ± 20.34, and 194 ± 21.29, respectively. We performed several analyses to (i) identify large-scale chromosomal and segmental duplication and deletion events, (ii) broadly characterize genomic patterns of CNVs, and (iii) identify CNVs that are differentiated between VGII subgroups.

**Fig. 2:**
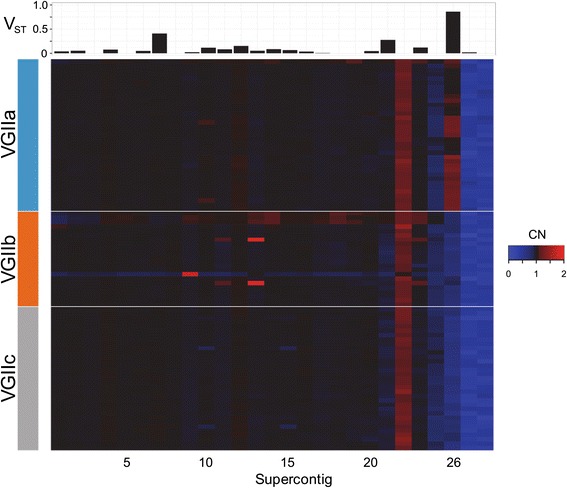
Supercontig copy number estimates between VGII subgroups. In the heatmap *blue*, *black*, and *red* represent supercontig copy numbers of 0, 1, and 2, respectively. Supercontigs and individuals and are represented on the X-axis and Y-axis, respectively. The V_ST_ value between VGIIa, VGIIb, and VGIIc subpopulations is reported for each supercontig (*upper* panel). Supercontig 26 present at a higher copy number in VGIIa individuals. Individual supercontig duplications can be seen for three isolates in VGIIb

Aneuploidy has been previously linked to variance in fungal pathogenicity, thus, we estimated CN for each supercontig (Fig. 2) [29, 30, 63, 64]. The vast majority of supercontigs were present at a CN of ~1; however, some supercontigs were present at higher or lower CN between subgroups. We calculated V_ST_ between CN estimates of entire supercontigs to identify those that differed between VGII subgroups. The average V_ST_ value for supercontigs was 0.097 suggesting most supercontigs were equally represented between subgroups and that variation is similarly partitioned between VGII subpopulations rather than within individual subpopulations. However, supercontig 26 was found at greater CN in VGIIa (1.14 ± 0.111 CN) compared to VGIIb (0.75 ± 0.068 CN) and VGIIc (0.67 ± 0.066 CN) (V_ST_ = 0.861) (Fig. 2). Supercontig 26 is one of the smallest supercontigs (15,500 bp) in the R265 reference genome and contains only one short uncharacterized gene (CNBG_9678) with no annotated protein domains. We also identified three individuals with atypical supercontig CNs. Two VGIIb isolates had an estimated CN of 1.87 and 1.84 for supercontig 13, and another VGIIb isolate had an estimated copy number of 1.75 for supercontig 9. A large scale duplication on supercontig 13 has been previously reported in a clinically isolated VGII sample from France [37].

**Fig. 3:**
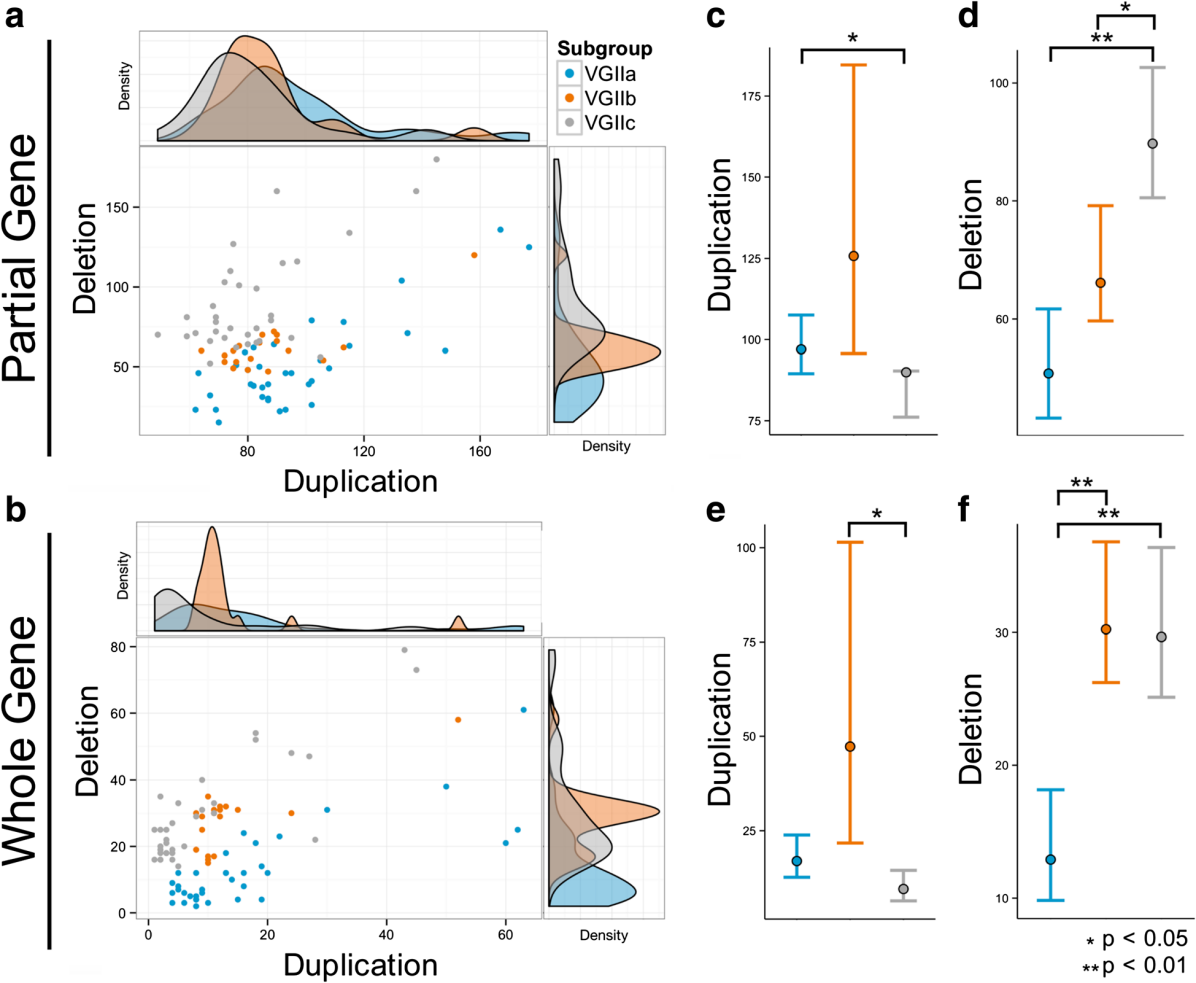
Genes overlapping with copy number variants across VGIIa, VGIIb, and VGIIc isolates. Scatter plots represent the number of duplications (X-axis) and deletions (Y-axis) that partially (**a**) and (**b**) entirely overlap genes, with marginal density distribution plots for each axis. **c–f** 95 % confidence intervals and VGIIa, VGIIb, and VGIIc subpopulation means were calculated using 1,000,000 bootstrap replicates. VGIIc has fewer partially duplicated genes than VGIIa (*p* = 0.034) (**c**) and more partially deleted genes than VGIIa (*p* = 0.006) and VGIIb (*p* = 0.012) (**e**). VGIIc has fewer whole duplicated genes than VGIIb (*p* = 0.039) (**d**), and VGIIa has fewer whole deleted genes than VGIIb (*p* <6e^−5^) and VGIIc (*p* <6e^−5^) (**f**). Three individuals from VGIIb with high frequencies of duplication events were removed for scaling in (**a**) and (**b**) but were retained for statistical analysis (**c–f**)

For high-resolution CNV prediction in each VGII isolate, we calculated integer CN for each 100 bp non-overlapping window in the genome using *Control-FREEC* [48]. To examine the potential functional effects of CNVs, we compared the frequency of genes for which CN variable regions partially or fully overlapped relative to the R265 reference genome (Fig. 3). VGIIa had the fewest partially deleted genes, VGIIb had the greatest partially deleted genes, and VGIIc had the fewest partially duplicated genes and the greatest partially deleted genes (Fig. 3a). The number of partially duplicated genes significantly differed between subgroups (Fisher-Pitman permutation test; *p* = 8.88e^−3^) and post-hoc pairwise comparisons revealed that VGIIc had fewer partially duplicated genes than VGIIa (*p* = 0.034) but not VGIIb (*p* >0.05) (Fig. 3c). Further, we found that the frequency of partially deleted genes was significantly different between VGII subgroups (Fisher-Pitman permutation test; *p* <2.2e^−16^) with post-hoc pairwise comparisons revealing that VGIIb had higher levels of partially deleted genes than VGIIa (*p* = 0.006) and VGIIb (*p* = 0.012) (Fig. 3d). We also found significant differences in the number of whole duplicated (*p* = 0.0051) and deleted (*p* <2e^−6^) genes between VGII subpopulations. VGIIb had significantly more duplicated genes than VGIIc (*p* = 0.039), while VGIIa had significantly fewer whole deleted genes compared with VGIIb (*p* <6e^−5^) and VGIIc (*p* <6e^−5^) (Fig. 3e and f). Reference genome bias might account for the lower frequency of CNVs in VGIIa since R265 is a member of the VGIIa subgroup.

### Identifying divergent CNVs between VGII subgroups

**Fig. 4:**
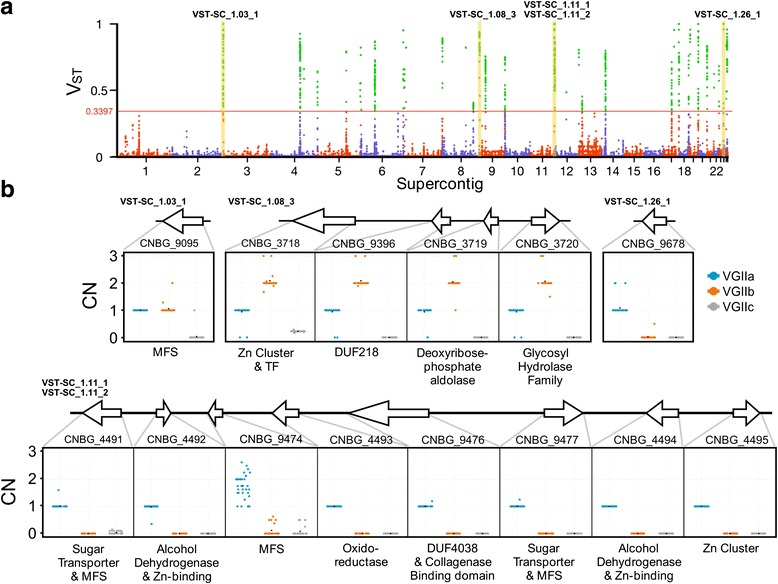
Loci with distinct subpopulation copy number profiles. **a** Manhattan plot of V_ST_ values (Y-axis) for each 100 bp bin (X-axis) in the *Cryptococcus gattii* genome. The *horizontal red* line represents the upper 99 % percentile cutoff. Points above this line are considered significantly differentiated CNVs. The five highest V_ST_ loci that contain genes are highlighted in *yellow* (supercontig 11 contains two of the five loci). **b** Gene copy number estimates for the five highest V_ST_ gene-containing loci. The gene ID and V_ST_ ID are reported for each locus. *Blue*, *orange*, and *gray* points represent VGIIa, VGIIb, and VGIIc isolates. Functional annotation (PFam domains or gene name) is provided below each plot

We hypothesized that CN profiles that segregated by VGII subgroups might contribute to the pathological differences between VGII subgroups. To identify these divergent CNV regions, we calculated V_ST_ for each 100 bp bin across the genome between VGIIa, VGIIb, and VGIIc subgroups [9]. Values near 0 represent no CN differentiation between subpopulations while values of 1 are indicative of fixed copy number differences between subpopulations. The average V_ST_ value for genomic bins where at least one isolate was copy number variable was 0.041, suggesting that the majority of CNV loci are not differentiated between VGII subpopulations. To identify highly differentiated CNVs, we examined loci in the upper 99 % percentile of V_ST_ values (V_ST_ ≥0.3397). Using this approach, we identified 67 loci harboring a total of 58 genes (Fig. 4a, Additional file 3). One of the highest V_ST_ regions (0.885) is present on supercontig 8, encompasses 10,900 bp, and contains four genes. Each gene in this locus is present near one copy in VGIIa, two copies in VGIIb, and zero copies in VGIIc. Two of the high V_ST_ loci are separated by 100 bp on supercontig 11 (Fig. 4b). The total length of these two neighboring high V_ST_ loci is 20.5 Kb and the region contains eight genes. All genes within this region are present as a single copy in VGIIa but absent in VGIIb and VGIIc. This region has been reported previously using a different computational approach and reinforces the efficacy of our methodology [33].

Lastly, we compiled a list of candidate genes associated with *Cryptococcus* and fungal pathogenicity from the literature and examined their CNV profiles [65–89]. A comprehensive suite of genes empirically related to virulence in *C. gattii* or *C. neoformans* (53 total genes) and chitin synthase genes (10 total genes) revealed very few deletion or duplication events. However, several potential virulence associated genes were found in high V_ST_ regions and are discussed later. We did identify CNVs in several genes related to transport activity. The majority of high V_ST_ transporters show the same pattern in which VGIIa isolates retain a single copy, while VGIIb and VGIIc isolates have experienced deletions (*CNBG_9639*, *CNBG_2482*, *CNBG_4491*, *CNBG_2944*) (Additional file 3).

### Functional analysis of CNV genes

We performed enrichment analysis of genes overlapping CNVs to broadly characterize their functional associations. First, we identified orthologs between *C. neoformans* and *C. gattii* using the Reciprocal Best BLAST Hit (RBBH) approach [57, 58]. *C. neoformans* GO annotation was assigned to *C. gattii* genes with one-to-one orthology [56]. We performed GO term enrichment analysis on all genes in the high V_ST_ CNV regions, on the genes overlapping CNV regions within each VGII subpopulation, and on the genes overlapping CNV regions across all VGII subpopulations. We independently analyzed genes that were (i) deleted partially, (ii) deleted entirely, (iii) duplicated partially, and (iv) duplicated entirely because each type of mutation cannot be categorized as “functional” or “nonfunctional” without experimental validation. For instance, partial gene deletions and duplications may result in loss of function, but may also be adaptive [90–92]. No GO terms were significantly enriched in the combined VGII subpopulations when entire deleted genes, partially deleted genes, or entire duplicated gene sets were analyzed. However, in the gene set composed of CNV regions that partially overlap genes across the combined VGII samples, we identified several enriched GO terms (for a full list of enriched GO terms see Additional file 4). The top three enriched GO terms in the biological processes category were *transmembrane transport* (*p* = 1.7e^−4^), *methylation* (*p* = 2.4e^−3^), *transport* (*p* = 5.4e^−3^), the top three enriched GO terms in the molecular function category were *substrate-specific transmembrane transporter activity* (*p* = 3.41e^−5^), *transmembrane transporter activity* (*p* = 3.98e^−5^), *receptor activity* (*p* = 1.15e^−2^), and the top three GO terms in the cellular component category were *proton-transporting V-type ATPase, V1 domain* (*p* = 1.69e^−4^), *proton-transporting V-type ATPase complex* (*p* = 1.4.3e^−3^), and *proton-transporting two-sector ATPase complex, catalytic domain* (*p* = 2.2e^−3^) (Additional file 4).

**Fig. 5:**
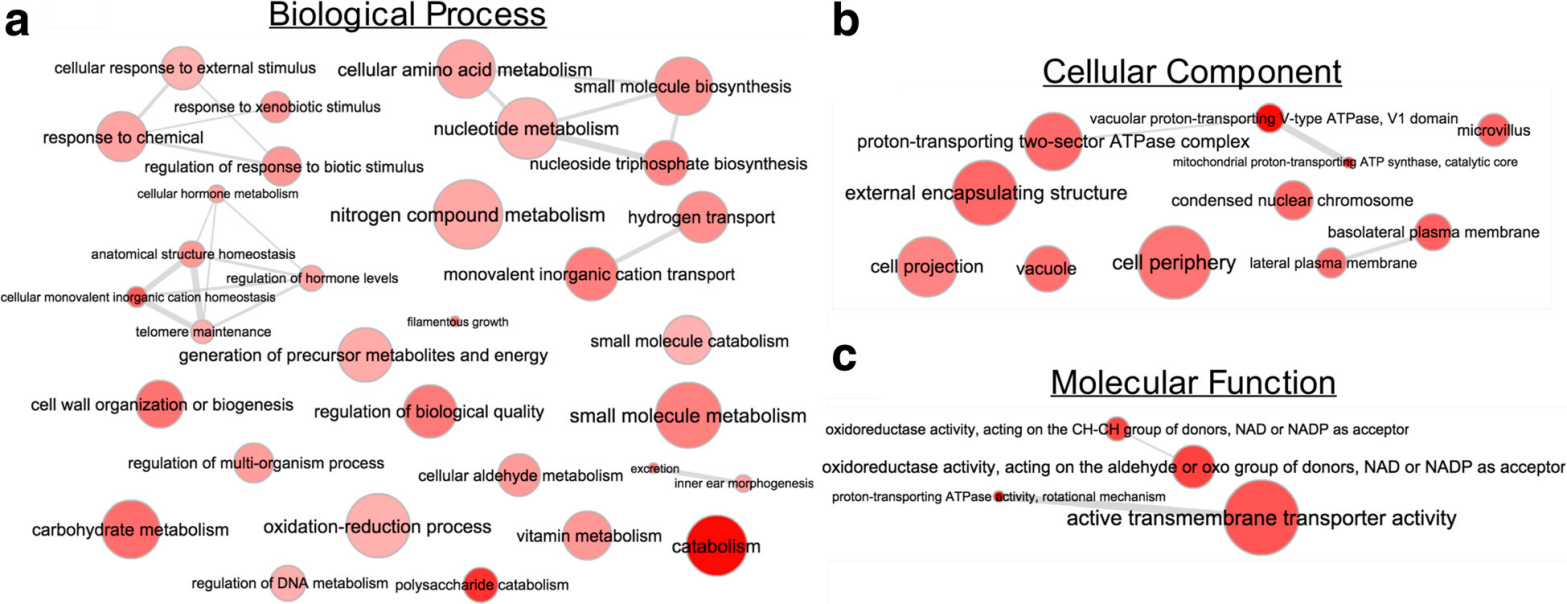
Functional enrichment of high V_ST_ loci. Gene Ontology (GO) enrichment based on PFam domains present in the genes overlapping high V_ST_ loci. Enrichment analysis was conducted via *dcGO* and is independently reported for Biological Processes (**a**), Cellular Component (**b**), and Molecular Function (**c**) [66]. Terms with *p*-values <0.01 are depicted. The shading of red represents *p*-value, with dark red corresponding to the lowest *p*-value. The area of each circle is relative to the number of terms falling into the respective GO category. *Grey* lines connect related GO terms and the thickness of the line indicated the degree of relatedness

We also examined the enrichment of PFam domains using dcGO to provide an additional level of resolution for functional analysis of high V_ST_ genes [61, 93]. Within the biological processes category, we observed enrichment of protein domains associated with primary metabolism (Fig. 5a). In particular, several of these categories are associated with fundamental processes such as *carbohydrate metabolic process* (*p* = 1.99e^−5^), *telomere maintenance* (*p* = 2.04e^−3^), and *mitochondrial proton-transporting ATP synthase, catalytic core* (*p* = 4.83e^−7^). Additionally, we identified several terms that are associated with environmental sensing, including *regulation of response to biotic stimulus* (*p* = 4.86e^−4^) and *response to xenobiotic stimulus* (*p* = 5.59e^−4^). Analysis of terms related to the cellular component category revealed several terms associated with the cell surface, including *external encapsulating structure* (*p* = 5.82e^−4^), *microvillus* (*p* = 4.3e^−4^) and *proton-transporting two-sector ATPase complex* (*p* = 6.72e^−4^) (Fig. 5b, Additional file 5). We also identified an enrichment of transport-related domains including *active transmembrane transporter activity* (*p* = 8.3e^−4^), *cation-transporting ATPase activity* (*p* = 9.56e^−5^), and *proton-transporting ATPase activity, rotational mechanism* (*p* = 3.31e^−6^) (Fig. 5c, Additional file 5).

## Discussion

We have conducted the first genome-wide population genomic analysis of *C. gattii* CN variation. Our high-resolution analysis revealed SVs ranging from 100 to 365,300 bp (Figs. 2, 3 and 4) encompassing ~1–2 % of the genome. The proportion of the genome affected by CNVs are in agreement to levels found in *Homo sapiens* (~5 %), *Danio rerio* (~4.5 %), *Sus scrofa domesticus* (1.5 %), *Arabidopsis thaliana* (0.75 %), and the yeasts *S. cerevisiae* (1.2 %) and *S. paradoxus* (3.5 %) [13, 26, 31, 94, 95].

We were primarily interested in identifying differentiated CNVs between the VGIIa, VGIIb, and VGIIc subpopulations given their close phylogenetic relationship but distinct pathological phenotypes. We calculated the CN specific population statistic V_ST_ to identify CN profiles that differed between VGII subpopulations (Fig. 4). Selection can cause patterns of CNV divergence between populations, however, other population genetic process, such as genetic drift and gene flow can also contribute to CNV profiles that are stratified by population. Nonetheless, the V_ST_ approach has offered insights into phenotypic differences between populations [31, 51, 96–98]. For instance, high V_ST_ loci in South African Nguni cattle revealed CN variable genes involved in parasite resistance, body size, and fertility that may account for phenotypic differences between breeds [98].

In our analysis, one of the highest V_ST_ values was obtained for a ~20 Kb region of supercontig 11 (VST-SC_1.11_1 and VST-SC_1.11_2) that contained 8 genes associated with sugar transport, alcohol dehydrogenase activity, and collagenase activity (Fig. 4b). In VGIIa the locus is present at a single copy, while deleted from the VGIIb and VGIIc isolates. This locus and pattern had been previously discovered through *de novo* genome assembly, *in silico* gene prediction, and BLAT score ratio analysis between VGIIa, VGIIb, and VGIIc isolates [33]. Another high scoring V_ST_ region included a four gene, 10.9 Kb region on supercontig 8 (VST-SC_1.26_1) with a CN of 1, 2, and 0 in the VGIIa, VGIIb, and VGIIc sub-populations, respectively (Fig. 5b). This locus included a putative transcription factor with a Zinc cluster domain, a glycosyl hydrolase family gene, and a deoxyribose-phosphate aldolase. In *Staphylococcus pneumoniae* a glycosyl hydrolase encoding gene (*GHIP*) is involved in host-cell invasion and knockout mutants exhibit reduced capacity to colonize mouse tissue [99]. In *Toxoplasma gondii*, a deoxyribose-phosphate aldolase plays an essential role in pathogenicity by mediating host-cell invasion, and providing energy during the invasion process [100].

Enrichment analysis of genes in high V_ST_ regions revealed cohesive networks of functional terms potentially linked to *C. gattii* pathogenicity, including many transport related GO terms (Fig. 5, Additional files 4 and 5). Transporter encoding genes are often associated with fungal virulence because of their ability to export fungicides from the cell [101]. Differences in fluconazole minimum inhibitory concentration within and between VGII subgroups have been observed, but have not been attributed to target gene (*ERG11)* expression or genetic variation [65, 67]. Rather, fluconazole resistance in *C. neoformans* and *C. gattii* appears to be driven by up-regulation of the ABC transporter encoding genes *ARF1*, *ARF2,* and *MDR1* [65, 102, 103]. Genes encoding a diversity of other transporters also contribute to *Cryptococcus* pathogenicity through such mechanisms as micronutrient scavenging and sugar transport for capsule formation [104–106]. Several Major Facilitator Superfamily (MFS) domains were predicted in the high V_ST_ genes including *CNBG_4491* (VGIIa: one copy, and VGIIb and VGIIc: zero copies), *CNBG_9477* (VGIIa: one copy, and VGIIb and VGIIc: zero copies), and *CNBG_9094* (VGIIa and VGIIb: two copies, and VGIIc: one copy).

The anti-phagocytic capsule is one of the defining virulence factors in *C. neoformans* and *C. gattii* and is primarily composed of a lattice of glucuronoxylomannan, glucuronoxylomannogalactan, and mannoproteins [34]. We observed the enrichment of several GO terms in the high V_ST_ genes that might be associated with the capsule, including *polysaccharide catabolism* and *external encapsulating structure* (Fig. 5). For instance, *CNBG_1084* contains a fibronectin type III-like domain. This domain can interact with the *ARF1* protein, which, along with its contribution to fluconazole resistance, is also involved in vesicle transport and influences capsule size [107, 108]. In VGIIc, the CNV found in *CNBG_1084* results in a ~600 bp deletion in the 3′ end of the gene and overlaps with the fibronectin type III-like domain and the stop codon. We also identified a highly differentiated 100 bp CNV (V_ST_ = 0.94) located in the 3′ end of a histone deacetylase (HDAC) encoding gene (*CNBG_5703*) (Additional file 3). This CNV did not overlap with the HDAC domain but did overlap with the stop codon (VGIIa: one copy, and VGIIb and VGIIc: zero copies). Through phenotypic screening of >1000 targeted gene deletion mutants in *C. neoformans*, Liu *et al.* [109] demonstrated that HDAC encoding genes contribute to capsule formation and influence infectivity.

CNVs can affect phenotype through changes in gene dosage, which most commonly correspond to alterations in gene expression [20, 22, 110–112]. Using a high resolution *in silico* approach, we have identified 67 CN variable regions in the *C. gattii* genome that contain 58 genes and are highly differentiated by VGII subgroup (Fig. 4, Additional file 3). These loci may account for some of the observed pathological differences between VGII subgroups. Moving forward, it will be essential to experimentally evaluate the function roles of these genes and to assess the regulatory impact of CNVs. Recent developments in molecular tools, such as RNA interference and CRISPR-Cas9 genome editing, as well as a murine model of *C. gattii* meningoencephalitis, will aid in assessing the function of *C. gattii* CNV gene candidates [113, 114].

## Conclusion

We identified 90 *C. gattii* isolates corresponding to the subpopulations responsible for the deadly outbreaks in the North American Pacific North West. Within these isolates, we bioinformatically predicted CNVs and uncovered loci that display subpopulation specific patterns of variation. Many of these CNV differentiated loci harbored genes that were enriched for metabolic, transport, and capsule composition associated functions. These genes represent novel candidates that may explain some of the pathological differences between VGII subgroups. Further functional investigation of candidate genes is needed to better understand the impact of copy number variation on *C. gattii* virulence.

## Additional files

Additional file 1: NCBI Sequence Read Archive run accession numbers for whole-genome Illumina data of the 212 *Cryptococcus gattii* isolates. VGII subgroup classification based on *Structure* and *DAPC* analysis (where *K* = 8) is provided where applicable. (XLSX 13 kb) Additional file 2: Individual copy number estimates and V_ST_ for each 100 bp bin relative to the *Cryptococcus gattii* R265 genome. (XLSX 49905 kb) Additional file 3: High V_ST_ regions with genome coordinates and gene identifiers relative to *Cryptococcus gattii* R265. (XLSX 15 kb) Additional file 4: Gene Enrichment Analysis of GO terms for CNV regions that partially overlap genes. Enrichment analysis is based on *Cryptococcus neoformans* annotation for genes with 1:1 orthology between *C. neoformans* and *C. gattii*. (XLSX 12 kb) Additional file 5: Functional enrichment analysis GO terms for high VST genes based on PFam domains. (XLSX 12 kb)

## Abbreviations

BIC: Bayesian information criterion
bp: Base pairs
CN: Copy number
CNV: Copy number variation
CV: Structural variation
DAPC: Discriminant analysis of principal components
GO: Gene ontology
Kb: Kilobases
LASSO: Least absolute shrinkage and selection operator
MCMC: Markov Chain Monte Carlo
MFS: Major facilitator superfamily
NMRV: Normalized mapped read value
nt: Nucleotides
PNW: Pacific Northwest
RBBH: Reciprocal best BLAST hit
SNPs: Single nucleotide polymorphisms

## References

[1] Feuk L, Carson AR, Scherer SW (2006). Structural variation in the human genome. Nat Rev Genet.

[2] Campbell CD, Eichler EE (2013). Properties and rates of germline mutations in humans. Trends Genet.

[3] Keith N, Tucker AE, Jackson CE, Sung W, Lucas Lledo JI, Schrider DR, Schaack S, Dudycha JL, Ackerman M, Younge AJ (2016). High mutational rates of large-scale duplication and deletion in Daphnia pulex. Genome Res.

[4] Lupski JR, Stankiewicz P (2005). Genomic disorders: molecular mechanisms for rearrangements and conveyed phenotypes. PLoS Genet.

[5] Lee C, Iafrate AJ, Brothman AR (2007). Copy number variations and clinical cytogenetic diagnosis of constitutional disorders. Nat Genet.

[6] Kazazian HH, Moran JV (1998). The impact of L1 retrotransposons on the human genome. Nat Genet.

[7] Mkrtchyan H, Gross M, Hinreiner S, Polytiko A, Manvelyan M, Mrasek K, Kosyakova N, Ewers E, Nelle H, Liehr T (2010). The human genome puzzle - the role of copy number variation in somatic mosaicism. Curr Genomics.

[8] Sudmant PH, Kitzman JO, Antonacci F, Alkan C, Malig M, Tsalenko A, Sampas N, Bruhn L, Shendure J, Genomes P (2010). Diversity of human copy number variation and multicopy genes. Science.

[9] Redon R, Ishikawa S, Fitch KR, Feuk L, Perry GH, Andrews TD, Fiegler H, Shapero MH, Carson AR, Chen W (2006). Global variation in copy number in the human genome. Nature.

[10] Bickhart DM, Hou Y, Schroeder SG, Alkan C, Cardone MF, Matukumalli LK, Song J, Schnabel RD, Ventura M, Taylor JF (2012). Copy number variation of individual cattle genomes using next-generation sequencing. Genome Res.

[11] Ghosh S, Qu Z, Das PJ, Fang E, Juras R, Cothran EG, McDonell S, Kenney DG, Lear TL, Adelson DL (2014). Copy number variation in the horse genome. PLoS Genet.

[12] Wang Y, Xiong G, Hu J, Jiang L, Yu H, Xu J, Fang Y, Zeng L, Xu E, Xu J (2015). Copy number variation at the GL7 locus contributes to grain size diversity in rice. Nat Genet.

[13] Zarrei M, MacDonald JR, Merico D, Scherer SW (2015). A copy number variation map of the human genome. Nat Rev Genet.

[14] Tang YC, Amon A (2013). Gene copy-number alterations: a cost-benefit analysis. Cell.

[15] Sjodin P, Jakobsson M (2012). Population genetic nature of copy number variation. Methods Mol Biol.

[16] Henrichsen CN, Chaignat E, Reymond A (2009). Copy number variants, diseases and gene expression. Hum Mol Genet.

[17] Zhang H, Zeidler AF, Song W, Puccia CM, Malc E, Greenwell PW, Mieczkowski PA, Petes TD, Argueso JL (2013). Gene copy-number variation in haploid and diploid strains of the yeast Saccharomyces cerevisiae. Genetics.

[18] Teshima KM, Innan H (2012). The coalescent with selection on copy number variants. Genetics.

[19] Zhang F, Gu W, Hurles ME, Lupski JR (2009). Copy number variation in human health, disease, and evolution. Annu Rev Genomics Hum Genet.

[20] Perry GH, Dominy NJ, Claw KG, Lee AS, Fiegler H, Redon R, Werner J, Villanea FA, Mountain JL, Misra R (2007). Diet and the evolution of human amylase gene copy number variation. Nat Genet.

[21] Nair S, Miller B, Barends M, Jaidee A, Patel J, Mayxay M, Newton P, Nosten F, Ferdig MT, Anderson TJ (2008). Adaptive copy number evolution in malaria parasites. PLoS Genet.

[22] Cook DE, Lee TG, Guo X, Melito S, Wang K, Bayless AM, Wang J, Hughes TJ, Willis DK, Clemente TE (2012). Copy number variation of multiple genes at Rhg1 mediates nematode resistance in soybean. Science.

[23] Fadista J, Thomsen B, Holm LE, Bendixen C (2010). Copy number variation in the bovine genome. BMC Genomics.

[24] Berglund J, Nevalainen EM, Molin AM, Perloski M, Consortium L, Andre C, Zody MC, Sharpe T, Hitte C, Lindblad-Toh K (2012). Novel origins of copy number variation in the dog genome. Genome Biol.

[25] Locke ME, Milojevic M, Eitutis ST, Patel N, Wishart AE, Daley M, Hill KA (2015). Genomic copy number variation in Mus musculus. BMC Genomics.

[26] Paudel Y, Madsen O, Megens HJ, Frantz LA, Bosse M, Bastiaansen JW, Crooijmans RP, Groenen MA (2013). Evolutionary dynamics of copy number variation in pig genomes in the context of adaptation and domestication. BMC Genomics.

[27] Qutob D, Tedman-Jones J, Dong S, Kuflu K, Pham H, Wang Y, Dou D, Kale SD, Arredondo FD, Tyler BM (2009). Copy number variation and transcriptional polymorphisms of Phytophthora sojae RXLR effector genes Avr1a and Avr3a. PLoS One.

[28] Selmecki A, Forche A, Berman J (2006). Aneuploidy and isochromosome formation in drug-resistant Candida albicans. Science.

[29] Hu G, Wang J, Choi J, Jung WH, Liu I, Litvintseva AP, Bicanic T, Aurora R, Mitchell TG, Perfect JR (2011). Variation in chromosome copy number influences the virulence of Cryptococcus neoformans and occurs in isolates from AIDS patients. BMC Genomics.

[30] Farrer RA, Henk DA, Garner TW, Balloux F, Woodhams DC, Fisher MC (2013). Chromosomal copy number variation, selection and uneven rates of recombination reveal cryptic genome diversity linked to pathogenicity. PLoS Genet.

[31] Brown GD, Denning DW, Gow NA, Levitz SM, Netea MG, White TC (2012). Hidden killers: human fungal infections. Sci Transl Med.

[32] Byrnes EJ, Li W, Lewit Y, Ma H, Voelz K, Ren P, Carter DA, Chaturvedi V, Bildfell RJ, May RC (2010). Emergence and pathogenicity of highly virulent Cryptococcus gattii genotypes in the northwest United States. PLoS Pathog.

[33] Engelthaler DM, Hicks ND, Gillece JD, Roe CC, Schupp JM, Driebe EM, Gilgado F, Carriconde F, Trilles L, Firacative C (2014). Cryptococcus gattii in North American Pacific Northwest: whole-population genome analysis provides insights into species evolution and dispersal. MBio.

[34] Bielska E, May RC (2016). What makes Cryptococcus gattii a pathogen?. FEMS Yeast Res.

[35] Farrer RA, Desjardins CA, Sakthikumar S, Gujja S, Saif S, Zeng Q, Chen Y, Voelz K, Heitman J, May RC (2015). Genome evolution and innovation across the four major lineages of Cryptococcus gattii. MBio.

[36] Springer DJ, Phadke S, Billmyre B, Heitman J (2012). Cryptococcus gattii, no longer an accidental pathogen?. Curr Fungal Infect Rep.

[37] Billmyre RB, Croll D, Li W, Mieczkowski P, Carter DA, Cuomo CA, Kronstad JW, Heitman J (2014). Highly recombinant VGII Cryptococcus gattii population develops clonal outbreak clusters through both sexual macroevolution and asexual microevolution. MBio.

[38] Hagen F, Ceresini PC, Polacheck I, Ma H, van Nieuwerburgh F, Gabaldon T, Kagan S, Pursall ER, Hoogveld HL, van Iersel LJ (2013). Ancient dispersal of the human fungal pathogen Cryptococcus gattii from the Amazon rainforest. PLoS One.

[39] Gillece JD, Schupp JM, Balajee SA, Harris J, Pearson T, Yan Y, Keim P, DeBess E, Marsden-Haug N, Wohrle R (2011). Whole genome sequence analysis of Cryptococcus gattii from the Pacific Northwest reveals unexpected diversity. PLoS One.

[40] Langmead B, Salzberg SL (2012). Fast gapped-read alignment with Bowtie 2. Nat Methods.

[41] Li H, Handsaker B, Wysoker A, Fennell T, Ruan J, Homer N, Marth G, Abecasis G, Durbin R, Genome Project Data Processing S (2009). The Sequence Alignment/Map format and SAMtools. Bioinformatics.

[42] Sims D, Sudbery I, Ilott NE, Heger A, Ponting CP (2014). Sequencing depth and coverage: key considerations in genomic analyses. Nat Rev Genet.

[43] Koboldt DC, Zhang Q, Larson DE, Shen D, McLellan MD, Lin L, Miller CA, Mardis ER, Ding L, Wilson RK (2012). VarScan 2: somatic mutation and copy number alteration discovery in cancer by exome sequencing. Genome Res.

[44] Pritchard JK, Stephens M, Donnelly P (2000). Inference of population structure using multilocus genotype data. Genetics.

[45] Evanno G, Regnaut S, Goudet J (2005). Detecting the number of clusters of individuals using the software STRUCTURE: a simulation study. Mol Ecol.

[46] Earl DA, vonHoldt BM (2011). STRUCTURE HARVESTER: a website and program for visualizing STRUCTURE output and implementing the Evanno method. Conserv Genet Resour.

[47] Jombart T, Ahmed I (2011). Adegenet 1.3-1: new tools for the analysis of genome-wide SNP data. Bioinformatics.

[48] Boeva V, Popova T, Bleakley K, Chiche P, Cappo J, Schleiermacher G, Janoueix-Lerosey I, Delattre O, Barillot E (2012). Control-FREEC: a tool for assessing copy number and allelic content using next-generation sequencing data. Bioinformatics.

[49] Li W, Olivier M (2013). Current analysis platforms and methods for detecting copy number variation. Physiol Genomics.

[50] Boeva V, Zinovyev A, Bleakley K, Vert JP, Janoueix-Lerosey I, Delattre O, Barillot E (2011). Control-free calling of copy number alterations in deep-sequencing data using GC-content normalization. Bioinformatics.

[51] Pezer Z, Harr B, Teschke M, Babiker H, Tautz D (2015). Divergence patterns of genic copy number variation in natural populations of the house mouse (Mus musculus domesticus) reveal three conserved genes with major population-specific expansions. Genome Res.

[52] Hothorn T, Hornik K, van de Wiel MA, Zeileis A (2008). Implementing a class of permutation tests: the coin package. J Stat Softw.

[53] Canty A, Ripley B (2015). boot: Bootstrap R (S-Plus) functions. R package version 1.3-17.

[54] Davison ACH, Hinkley DV (1997). Bootstrap Methods and Their Applications.

[55] Hervé M (2015). RVAideMemoire: diverse basic statistical and graphical functions. R package version 0.9-50.

[56] Chen Y, Toffaletti DL, Tenor JL, Litvintseva AP, Fang C, Mitchell TG, McDonald TR, Nielsen K, Boulware DR, Bicanic T (2014). The Cryptococcus neoformans transcriptome at the site of human meningitis. MBio.

[57] Salichos L, Rokas A (2011). Evaluating ortholog prediction algorithms in a yeast model clade. PLoS One.

[58] Altschul SF, Gish W, Miller W, Myers EW, Lipman DJ (1990). Basic local alignment search tool. J Mol Biol.

[59] Jones P, Binns D, Chang HY, Fraser M, Li W, McAnulla C, McWilliam H, Maslen J, Mitchell A, Nuka G (2014). InterProScan 5: genome-scale protein function classification. Bioinformatics.

[60] Zheng Q, Wang XJ (2008). GOEAST: a web-based software toolkit for Gene Ontology enrichment analysis. Nucleic Acids Res.

[61] Fang H, Gough J (2013). A domain-centric solution to functional genomics via dcGO predictor. BMC Bioinformatics.

[62] Supek F, Bosnjak M, Skunca N, Smuc T (2011). REVIGO summarizes and visualizes long lists of gene ontology terms. PLoS One.

[63] Raffaele S, Farrer RA, Cano LM, Studholme DJ, MacLean D, Thines M, Jiang RH, Zody MC, Kunjeti SG, Donofrio NM (2010). Genome evolution following host jumps in the Irish potato famine pathogen lineage. Science.

[64] Refsnider JM, Poorten TJ, Langhammer PF, Burrowes PA, Rosenblum EB (2015). Genomic correlates of virulence attenuation in the deadly amphibian Chytrid fungus, Batrachochytrium dendrobatidis. G3 (Bethesda).

[65] Basso LR, Gast CE, Bruzual I, Wong B (2015). Identification and properties of plasma membrane azole efflux pumps from the pathogenic fungi Cryptococcus gattii and Cryptococcus neoformans. J Antimicrob Chemother.

[66] Schneider Rde O, Fogaca Nde S, Kmetzsch L, Schrank A, Vainstein MH, Staats CC (2012). Zap1 regulates zinc homeostasis and modulates virulence in Cryptococcus gattii. PLoS One.

[67] Gast CE, Basso LR, Bruzual I, Wong B (2013). Azole resistance in Cryptococcus gattii from the Pacific Northwest: investigation of the role of ERG11. Antimicrob Agents Chemother.

[68] Feder V, Kmetzsch L, Staats CC, Vidal-Figueiredo N, Ligabue-Braun R, Carlini CR, Vainstein MH (2015). Cryptococcus gattii urease as a virulence factor and the relevance of enzymatic activity in cryptococcosis pathogenesis. FEBS J.

[69] Chen YL, Lehman VN, Lewit Y, Averette AF, Heitman J (2013). Calcineurin governs thermotolerance and virulence of Cryptococcus gattii. G3 (Bethesda).

[70] Schneider Rde O, Diehl C, dos Santos FM, Piffer AC, Garcia AW, Kulmann MI, Schrank A, Kmetzsch L, Vainstein MH, Staats CC (2015). Effects of zinc transporters on Cryptococcus gattii virulence. Sci Rep.

[71] Shimizu K, Imanishi Y, Toh-e A, Uno J, Chibana H, Hull CM, Kawamoto S (2014). Functional characterization of PMT2, encoding a protein-O-mannosyltransferase, in the human pathogen Cryptococcus neoformans. Fungal Genet Biol.

[72] Ngamskulrungroj P, Himmelreich U, Breger JA, Wilson C, Chayakulkeeree M, Krockenberger MB, Malik R, Daniel HM, Toffaletti D, Djordjevic JT (2009). The trehalose synthesis pathway is an integral part of the virulence composite for Cryptococcus gattii. Infect Immun.

[73] Ren P, Springer DJ, Behr MJ, Samsonoff WA, Chaturvedi S, Chaturvedi V (2006). Transcription factor STE12alpha has distinct roles in morphogenesis, virulence, and ecological fitness of the primary pathogenic yeast Cryptococcus gattii. Eukaryot Cell.

[74] Narasipura SD, Ault JG, Behr MJ, Chaturvedi V, Chaturvedi S (2003). Characterization of Cu, Zn superoxide dismutase (SOD1) gene knock-out mutant of Cryptococcus neoformans var. gattii: role in biology and virulence. Mol Microbiol.

[75] Chen SC, Muller M, Zhou JZ, Wright LC, Sorrell TC (1997). Phospholipase activity in Cryptococcus neoformans: a new virulence factor?. J Infect Dis.

[76] Ganendren R, Carter E, Sorrell T, Widmer F, Wright L (2006). Phospholipase B activity enhances adhesion of Cryptococcus neoformans to a human lung epithelial cell line. Microbes Infect.

[77] D’Souza CA, Alspaugh JA, Yue C, Harashima T, Cox GM, Perfect JR, Heitman J (2001). Cyclic AMP-dependent protein kinase controls virulence of the fungal pathogen Cryptococcus neoformans. Mol Cell Biol.

[78] Bahn YS, Kojima K, Cox GM, Heitman J (2006). A unique fungal two-component system regulates stress responses, drug sensitivity, sexual development, and virulence of Cryptococcus neoformans. Mol Biol Cell.

[79] Kronstad JW, Attarian R, Cadieux B, Choi J, D’Souza CA, Griffiths EJ, Geddes JM, Hu G, Jung WH, Kretschmer M (2011). Expanding fungal pathogenesis: Cryptococcus breaks out of the opportunistic box. Nat Rev Microbiol.

[80] Idnurm A, Walton FJ, Floyd A, Reedy JL, Heitman J (2009). Identification of ENA1 as a virulence gene of the human pathogenic fungus Cryptococcus neoformans through signature-tagged insertional mutagenesis. Eukaryot Cell.

[81] Idnurm A, Reedy JL, Nussbaum JC, Heitman J (2004). Cryptococcus neoformans virulence gene discovery through insertional mutagenesis. Eukaryot Cell.

[82] Waugh MS, Nichols CB, DeCesare CM, Cox GM, Heitman J, Alspaugh JA (2002). Ras1 and Ras2 contribute shared and unique roles in physiology and virulence of Cryptococcus neoformans. Microbiology.

[83] Kronstad JW, Hu G, Choi J (2011). The cAMP/Protein Kinase A pathway and virulence in Cryptococcus neoformans. Mycobiology.

[84] Giles SS, Stajich JE, Nichols C, Gerrald QD, Alspaugh JA, Dietrich F, Perfect JR (2006). The Cryptococcus neoformans catalase gene family and its role in antioxidant defense. Eukaryot Cell.

[85] Waterman SR, Hacham M, Hu G, Zhu X, Park YD, Shin S, Panepinto J, Valyi-Nagy T, Beam C, Husain S (2007). Role of a CUF1/CTR4 copper regulatory axis in the virulence of Cryptococcus neoformans. J Clin Invest.

[86] Bahn YS, Hicks JK, Giles SS, Cox GM, Heitman J (2004). Adenylyl cyclase-associated protein Aca1 regulates virulence and differentiation of Cryptococcus neoformans via the cyclic AMP-protein Kinase A cascade. Eukaryot Cell.

[87] Jung WH, Saikia S, Hu G, Wang J, Fung CK, D’Souza C, White R, Kronstad JW (2010). HapX positively and negatively regulates the transcriptional response to iron deprivation in Cryptococcus neoformans. PLoS Pathog.

[88] Okabayashi K, Hasegawa A, Watanabe T (2007). Microreview: capsule-associated genes of Cryptococcus neoformans. Mycopathologia.

[89] Mylonakis E, Idnurm A, Moreno R, El Khoury J, Rottman JB, Ausubel FM, Heitman J, Calderwood SB (2004). Cryptococcus neoformans Kin1 protein kinase homologue, identified through a Caenorhabditis elegans screen, promotes virulence in mammals. Mol Microbiol.

[90] Fidalgo M, Barrales RR, Ibeas JI, Jimenez J (2006). Adaptive evolution by mutations in the FLO11 gene. Proc Natl Acad Sci U S A.

[91] Katju V, Lynch M (2003). The structure and early evolution of recently arisen gene duplicates in the Caenorhabditis elegans genome. Genetics.

[92] Qian W, Zhang J (2014). Genomic evidence for adaptation by gene duplication. Genome Res.

[93] Finn RD, Coggill P, Eberhardt RY, Eddy SR, Mistry J, Mitchell AL, Potter SC, Punta M, Qureshi M, Sangrador-Vegas A (2016). The Pfam protein families database: towards a more sustainable future. Nucleic Acids Res.

[94] Xu X, Nagarajan H, Lewis NE, Pan S, Cai Z, Liu X, Chen W, Xie M, Wang W, Hammond S (2011). The genomic sequence of the Chinese hamster ovary (CHO)-K1 cell line. Nat Biotechnol.

[95] Bergstrom A, Simpson JT, Salinas F, Barre B, Parts L, Zia A, Nguyen Ba AN, Moses AM, Louis EJ, Mustonen V (2014). A high-definition view of functional genetic variation from natural yeast genomes. Mol Biol Evol.

[96] Xu L, Hou Y, Bickhart DM, Zhou Y, HA H e, Song J, Sonstegard TS, Van Tassell CP, Liu GE (2016). Population-genetic properties of differentiated copy number variations in cattle. Sci Rep.

[97] Sudmant PH, Mallick S, Nelson BJ, Hormozdiari F, Krumm N, Huddleston J, Coe BP, Baker C, Nordenfelt S, Bamshad M (2015). Global diversity, population stratification, and selection of human copy-number variation. Science.

[98] Wang MD, Dzama K, Hefer CA, Muchadeyi FC (2015). Genomic population structure and prevalence of copy number variations in South African Nguni cattle. BMC Genomics.

[99] Niu S, Luo M, Tang J, Zhou H, Zhang Y, Min X, Cai X, Zhang W, Xu W, Li D (2013). Structural basis of the novel S. pneumoniae virulence factor, GHIP, a glycosyl hydrolase 25 participating in host-cell invasion. PLoS One.

[100] Hassan IA, Wang S, Xu L, Yan R, Song X, Li X (2014). DNA vaccination with a gene encoding Toxoplasma gondii Deoxyribose Phosphate Aldolase (TgDPA) induces partial protective immunity against lethal challenge in mice. Parasit Vectors.

[101] Brown JS, Gilliland SM, Holden DW (2001). A Streptococcus pneumoniae pathogenicity island encoding an ABC transporter involved in iron uptake and virulence. Mol Microbiol.

[102] Sanguinetti M, Posteraro B, La Sorda M, Torelli R, Fiori B, Santangelo R, Delogu G, Fadda G (2006). Role of AFR1, an ABC transporter-encoding gene, in the in vivo response to fluconazole and virulence of Cryptococcus neoformans. Infect Immun.

[103] Posteraro B, Sanguinetti M, Sanglard D, La Sorda M, Boccia S, Romano L, Morace G, Fadda G (2003). Identification and characterization of a Cryptococcus neoformans ATP binding cassette (ABC) transporter-encoding gene, CnAFR1, involved in the resistance to fluconazole. Mol Microbiol.

[104] Cottrell TR, Griffith CL, Liu H, Nenninger AA, Doering TL (2007). The pathogenic fungus Cryptococcus neoformans expresses two functional GDP-mannose transporters with distinct expression patterns and roles in capsule synthesis. Eukaryot Cell.

[105] Xue C, Liu T, Chen L, Li W, Liu I, Kronstad JW, Seyfang A, Heitman J (2010). Role of an expanded inositol transporter repertoire in Cryptococcus neoformans sexual reproduction and virulence. MBio.

[106] Zaragoza O, Rodrigues ML, De Jesus M, Frases S, Dadachova E, Casadevall A (2009). The capsule of the fungal pathogen Cryptococcus neoformans. Adv Appl Microbiol.

[107] Walton FJ, Heitman J, Idnurm A (2006). Conserved elements of the RAM signaling pathway establish cell polarity in the basidiomycete Cryptococcus neoformans in a divergent fashion from other fungi. Mol Biol Cell.

[108] Paczkowski JE, Richardson BC, Strassner AM, Fromme JC (2012). The exomer cargo adaptor structure reveals a novel GTPase-binding domain. EMBO J.

[109] Liu OW, Chun CD, Chow ED, Chen C, Madhani HD, Noble SM (2008). Systematic genetic analysis of virulence in the human fungal pathogen Cryptococcus neoformans. Cell.

[110] Gibbons JG, Salichos L, Slot JC, Rinker DC, McGary KL, King JG, Klich MA, Tabb DL, McDonald WH, Rokas A (2012). The evolutionary imprint of domestication on genome variation and function of the filamentous fungus Aspergillus oryzae. Curr Biol.

[111] Handsaker RE, Van Doren V, Berman JR, Genovese G, Kashin S, Boettger LM, McCarroll SA (2015). Large multiallelic copy number variations in humans. Nat Genet.

[112] Maron LG, Guimaraes CT, Kirst M, Albert PS, Birchler JA, Bradbury PJ, Buckler ES, Coluccio AE, Danilova TV, Kudrna D (2013). Aluminum tolerance in maize is associated with higher MATE1 gene copy number. Proc Natl Acad Sci U S A.

[113] Skowyra ML, Doering TL (2012). RNA interference in Cryptococcus neoformans. Methods Mol Biol.

[114] Thompson GR, Wiederhold NP, Najvar LK, Bocanegra R, Kirkpatrick WR, Graybill JR, Patterson TF (2012). A murine model of Cryptococcus gattii meningoencephalitis. J Antimicrob Chemother.

